# Pharmacological and Biological Targeting of FGFR1 in Cancer

**DOI:** 10.3390/cimb46110783

**Published:** 2024-11-18

**Authors:** Shuai Fan, Yuxin Chen, Wenyu Wang, Wanting Xu, Mei Tian, Yuetong Liu, Yutong Zhou, Dan Liu, Qin Xia, Lei Dong

**Affiliations:** State Key Laboratory of Molecular Medicine and Biological Diagnosis and Treatment (Ministry of Industry and Information Technology), School of Life Science, Beijing Institute of Technology, Beijing 100081, China; 1120221191@bit.edu.cn (S.F.); cyx1036894604@163.com (Y.C.); 1120223086@bit.edu.cn (W.W.); xuwanting140910@163.com (W.X.); 1120222704@bit.edu.cn (M.T.); 1120230255@bit.edu.cn (Y.L.); 1120230191@bit.edu.cn (Y.Z.); uanea548326@163.com (D.L.)

**Keywords:** FGFR1, aberration, targeted therapeutics, TKI, antibody drugs, ligand traps

## Abstract

FGFR1 is a key member of the fibroblast growth factor receptor family, mediating critical signaling pathways such as RAS-MAPK and PI3K-AKT. which are integral to regulating essential cellular processes, including proliferation, differentiation, and survival. Alterations in FGFR1 can lead to constitutive activation of signaling pathways that drive oncogenesis by promoting uncontrolled cell division, inhibiting apoptosis, and enhancing the metastatic potential of cancer cells. This article reviews the activation mechanisms and signaling pathways of FGFR1 and provides a detailed exposition of the types of FGFR1 aberration. Furthermore, we have compiled a comprehensive overview of current therapies targeting FGFR1 aberration in cancer, aiming to offer new perspectives for future cancer treatments by focusing on drugs that address specific FGFR1 alterations.

## 1. Introduction

The human fibroblast growth factor family consists of 22 factors and five transmembrane receptors [1]. Of the 22 factors, 18 are secreted while four of them function exclusively within the cell [2]. The Fibroblast Growth Factor Receptor (FGFR) family consists of four main members: FGFR1, FGFR2, FGFR3, and FGFR4. These receptors are highly conserved across vertebrate species, highlighting their essential role in developmental and physiological processes. Structurally, each FGFR has an extracellular domain comprising three immunoglobulin-like (Ig-like) subdomains (D1, D2, and D3), a single transmembrane helix, and a cytoplasmic tyrosine kinase domain [3]. Four of the fibroblast growth factor receptors (FGFRs) possess intracellular protein–tyrosine kinase activity while the fifth (FGFRL1) has a short 105-residue intracellular non-enzymatic component [4]. Fibroblast growth factor receptors (FGFRs) are highly conserved, widely distributed transmembrane tyrosine kinase receptors [5]. Similar to other tyrosine kinase receptors, FGFR contains a bi-lobed kinase fold composed of two main lobes, the N-lobe and the C-lobe, which are separated by a cleft where ATP and substrates bind, thereby playing a crucial role in the receptor’s kinase activity and regulation [6]. They are involved in development, differentiation, cell survival, migration, angiogenesis and carcinogenesis [7]. FGFR gene alterations occur in a wide variety of cancers including those of the urinary bladder, breast, ovary, prostate, endometrium, lung, and stomach [2].

FGFR1 is located at 8p11.23 with a total length of 68,620 bases. It is one of the four members of the FGFR family and belongs to the receptor tyrosine kinase group [8]. FGFR1 is expressed in all tissues and organs throughout the body, especially in plasma, heart, and cerebrospinal fluid. FGFR1 is the most widely reported and studied gene among the FGFR family members, and it has been widely reported as an oncogene to promote tumor initiation and development, indicating its importance in tumorigenesis. The abnormal activation of FGFR1 is mainly associated with its genetic amplification, mutations, fusions, and rearrangements and is generally associated with high tumor aggressiveness, resistance to hormone therapy, poor differentiation, and adverse clinical outcomes. A study revealed that FGFR aberrations were present in 7.1% of cancers, with the majority of these alterations being gene amplifications (66%), followed by mutations (26%) and rearrangements (8%). Specifically, FGFR1, primarily affected by amplifications, was altered in 3.5% of the 4853 patients studied [9]. Different aberrations of FGFR1 in tumors have varying effects on the response to relevant drugs. This review will outline the structural features of FGFR1 and its signaling pathways, delineate the roles of different FGFR1 mutation types in tumorigenesis, and discuss the current pharmaceutical approaches applicable to specific FGFR1 aberration profiles.

## 2. FGFR1 Signaling

FGFR1 is a tyrosine kinase (TK) cell surface receptor, all of which are transmembrane proteins with multiple domains, including three extracellular immunoglobulin-like domains (I, II, and III, also referred to as D1/D2/D3), a transmembrane domain which is a single transmembrane helix (TM), and two intracellular tyrosine kinase domains (TK1 and TK2). There are many junction regions between the respective major domains that support the structure of FGFR1 [10].

The extracellular immunoglobulin-like domains II and III of FGFR1 and the junction regions between domains regulate the ligand-binding specificity of the FGFs [11]. For example, the acidic amino acid sequence-acid box (AB) between extracellular immunoglobulin-like domains I and II has been shown to co-inhibit the binding of FGFR to its receptor with domain I [12].The intracellular domain has TK activity. In the absence of FGF, the receptor exists as a monomer and the TK domain remains phosphorylated. In the presence of FGF, FGFR1 dimerization forms a functional 2FGF:2FGFR1 complex, activates kinases and forms transphosphorylation, which causes TK activation and downstream signaling.

**Figure 1:**
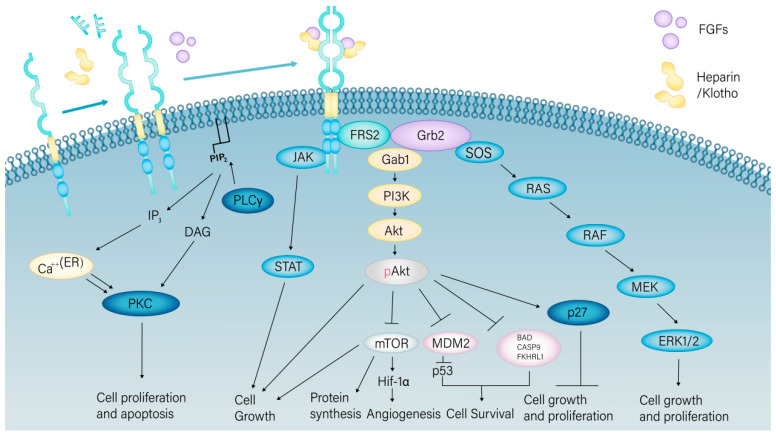
Activation modalities of FGFR1 and the cell signaling pathways it mediates. FGFR1 binds to FGFs with the assistance of Heparin (primarily aiding the FGF1, FGF4, FGF7, FGF8, FGF9 subfamilies) or Klotho (primarily assisting the FGF15/19 subfamilies), which alleviates its autoinhibitory state and activates its autophosphorylation. Subsequently, this activation triggers downstream pathways, such as PLCγ, RAS/MEK, MAPK/ERK, and PI3K/AKT, affecting cellular processes, including proliferation, survival, and differentiation.

In the presence of FGFs and Heparin/Klotho, FGFR1 kinases form dimers in the active DFG Asp-in configuration. FGF binds to the extracellular domains of FGFR, particularly the D2 and D3 domains and their linker regions, stabilizing the FGF–FGFR–heparan sulfate complex and promoting receptor dimerization. Upon dimerization, the kinase domain of FGFR transitions from an inactive conformation (DFG-Dout) to an active conformation (DFG-Din), where the aspartate residue in the DFG motif faces the activation site, facilitating the binding of ATP and substrates. Subsequently, multiple tyrosine residues in the activation segment (e.g., Y653 and Y654) undergo trans-phosphorylation, further enhancing kinase activity. Concurrently, the molecular brake within the FGFR domain, composed of the NEK triplet (asparagine, glutamate, and lysine), is released, breaking the hydrogen bond network and allowing the kinase domain to adopt an active conformation. The αC-helix shifts from an αCout to an αCin conformation, where the glutamate in the αC-helix forms a salt bridge with the lysine in the β3 strand, stabilizing the active site of the kinase. The alignment of the regulatory spine (R-Spine) and the stabilization of the catalytic spine (C-Spine) ensure ATP binding and participation in the catalytic reaction. This conformational change releases them from autoinhibition and enables autophosphorylation. Consequently, these activated kinases initiate downstream signaling cascades involving cytosolic adaptor proteins and activate intracellular pathways, such as RAS-MAPK, PI3K-AKT, phospholipase Cγ (PLCγ), and STAT [13] (Figure 1).

FGFR1 signaling plays a crucial role in numerous physiological processes, significantly impacting various stages of life from early embryonic development to adulthood. During embryogenesis, FGFR1 regulates essential cell behaviors, such as proliferation, differentiation, and migration, which are fundamental for proper tissue and organ formation. It orchestrates the complex morphogenesis of the nervous system, skeletal system, and cardiovascular structures, ensuring that organ systems develop with precise coordination and functionality [14]. In infancy and throughout postnatal development, FGFR1 remains indispensable for promoting growth and maintaining metabolic balance. It regulates adipocyte differentiation and influences glucose and lipid metabolism, thus playing a pivotal role in energy homeostasis [15]. FGFR1’s involvement in metabolic regulation is crucial for adapting to changing nutritional and energy demands as the body grows and matures [16]. Moreover, in adult tissues, FGFR1 signaling is integral to the body’s ability to repair and regenerate. It mediates cellular responses to injury by facilitating the proliferation and migration of fibroblasts, endothelial cells, and other cell types essential for tissue repair. This function is particularly evident in processes such as wound healing, where FGFR1 contributes to angiogenesis, collagen deposition, and tissue remodeling, thereby promoting effective recovery and restoration of tissue integrity [17]. Overall, FGFR1 serves as a master regulator that coordinates multiple physiological mechanisms across various stages of life. However, once FGFR1 alterations occur, the scenario changes significantly. Due to structural alterations and dysregulated expression of related factors, FGFR1 signaling can become aberrantly activated in tumor cells through multiple pathways. This aberrant activation ultimately enhances tumor cell invasiveness, and promotes proliferation, survival, tumor progression, and resistance to therapy. Additionally, FGFR1 mutations contribute to angiogenesis and immune evasion within the tumor microenvironment, further facilitating tumor growth and metastasis, thus complicating disease management [12,13].

## 3. FGFR1 Aberration and Its Role in Tumorigenesis and Its Development

**Figure 2:**
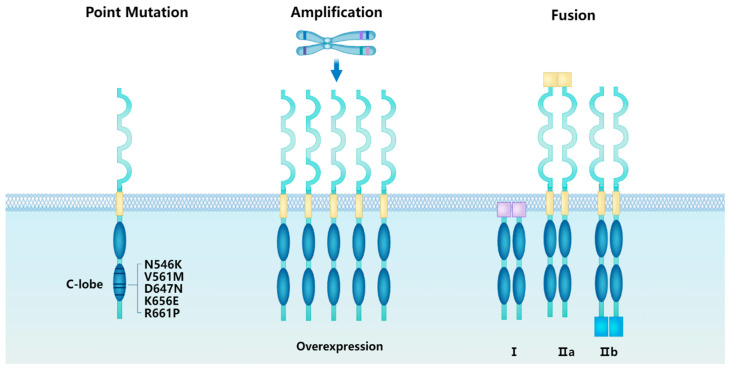
Aberration of FGFR1. FGFR1 mutations primarily include point mutations, gene amplifications, protein overexpression, and fusions and/or rearrangements. Point mutations chiefly encompass the kinase domain mutations N546K, V561M, D647N, K656E, and R661P, which have been observed in non-small cell lung cancer (NSCLC), head and neck squamous cell carcinoma, and bladder cancer. FGFR1 gene amplification may lead to overexpression of the FGFR1 protein, although protein overexpression is not necessarily solely caused by amplification. Amplification and overexpression of FGFR1 are predominantly seen in breast and gastric cancers. FGFR1 gene fusions, categorized into Type I and Type II, result in constitutive activation of FGFR1 and are frequently observed in squamous cell carcinoma, leukemia, and gliomas. Type I fusions involve non-receptor-type proteins with the N-terminus replaced, while Type II fusions can be further subdivided into Type IIa and Type IIb. Type IIa fusions involve receptor-type proteins with the N-terminus replaced, and Type IIb fusions involve receptor-type proteins with the C-terminus replaced.

Fibroblast growth factor receptor (FGFR) alterations drive oncogenesis in multiple tumor types. Constitutive FGFR signaling associated with gains in function in FGF receptors and abnormally high levels of autophosphorylation of FGFR1, play a role in tumor cell proliferation and growth, angiogenesis, and metastasis in various malignancies. Abnormal activation of FGFR1 occurs basically by three major mechanisms: gene amplification, translocation, or activating mutations (Figure 2).

### 3.1. Point Mutations

Although the frequency of FGFR1 point mutations in tumors has been reported to be relatively low, these mutations can induce constitutive activation of FGFR1, thereby serving as a driver of tumorigenesis. Moreover, such mutations may reduce the affinity of drugs that target FGFR1. FGFR1 point mutations result in constitutive activation of receptors and are drivers of tumors. The activation loop of the kinase is a critical regulatory region directly influencing the activity and function of the kinase protein. Mutations observed across various cancers, such as N546K, V561M, D647N, K656E and R661P, typically occur within this activation loop region. These mutations lead to elevated kinase activity, characterized by increased autophosphorylation levels or constitutive activation, promoting oncogenesis. In a study encompassing 4853 solid tumors, researchers identified five distinct FGFR1 mutations, including T141R, R445W, N546K, K656E, and G818R. N546K and K656E are reported as hotspots among FGFR1 mutations, significantly impacting FGFR1 activity. For instance, the N546K mutation accelerates the formation of monophosphorylated FGFR1 receptors 25-fold faster than the wild-type, leading to constitutive activation of FGFR1, while the K656E [9,18] mutation enhances autophosphorylation levels, resulting in constitutive activation of FGFR1 These mutations are observed across multiple diseases, notably in Encephalocraniocutaneous lipomatosis (ECCL), including conditions like scalp lesions, benign eye tumors, and central nervous system lipomas. Such aberrant kinase activities stimulate activation of the RAS–MAPK signaling pathway, closely associated with tumorigenesis [19]. Rivera et al. observed the R661P mutation in FGFR1 in Dysembryoplastic neuroepithelial tumor (DNET). The p.R661 residue is located in the C-terminal part of FGFR1’s activation loop. In the inhibited structure of FGFR1K, the side chain extends and forms a hydrogen bond with the carbonyl oxygen of G697, effectively blocking the binding site for substrate peptides. The R661P substitution disrupts this inhibitory hydrogen bond interaction, potentially altering the regulation of FGFR1’s kinase activity [20].Cowell et al. observed the V561M mutation in FGFR1 in leukemia and lymphomas. This mutation induces autophosphorylation of FGFR1. Additionally, the expression of FGFR1 D647N significantly enhances both individual and collective migration of cancer cells in vitro and in vivo, activating and mediating the PI3K/AKT signaling pathway. This pathway inhibition of apoptosis may promote tumorigenesis [21].

FGFR1 point mutations are responsible for the emergence of drug resistance in tumors. For FGFR1, the most well-known gatekeeper mutation is the V561M mutation. This mutation involves the substitution of valine (V) with methionine (M) at position 561 in the FGFR1 protein. The V561M mutation has been shown to cause resistance to several FGFR inhibitors by preventing these inhibitors from effectively binding to the kinase domain. Studies have shown that Lucitanib exhibits significantly reduced affinity for the V561M-mutant FGFR1, resulting in pronounced resistance, whereas AZD4547 maintains a high affinity for the V561M-mutant FGFR1 through a different binding mode. This suggests that the flexibility of the drug may be a key factor in overcoming resistance [19,22].

### 3.2. Amplification and/or Overexpression

FGFR1 amplification refers to an increase in the number of copies of the FGFR1 gene, which may lead to its overexpression, meaning that higher levels of FGFR1 protein are produced in the cells. This overexpression can enhance the FGFR1 signaling pathway, promoting cell proliferation, survival, and migration, which are key processes in cancer development and progression [23]. FGFR1 amplification or overexpression is prevalent in various tumor types, with the highest rates observed in lung squamous cell carcinoma, breast cancer, bladder cancer, head and neck squamous cell carcinoma, and endometrial cancer. Specifically, FGFR1 amplification occurs in approximately 19% of lung squamous cell carcinoma cases, 10% of breast cancer cases, 14% of bladder cancer cases, 10% of head and neck squamous cell carcinoma cases, and 7% of endometrial cancer cases [24].

FGFR1 amplification is closely associated with tobacco- or alcohol-related cancers. Studies have shown that FGFR1 gene amplification is present in 9.3% to 17.4% of patients with squamous head and neck cell carcinoma (HNSCC), while 11.8% of patients exhibit FGFR1 protein overexpression [25]. Further research by Kim et al. confirmed that high FGFR1 expression is more frequent with worsening histologic differentiation. FGFR1 amplification and overexpression are more commonly observed in hypopharyngeal and laryngeal squamous cell carcinoma (SCC) and have been identified as independent prognostic factors for disease-free survival (DFS). This suggests that alterations in FGFR1 are not only likely to contribute to the progression of these cancers but are also closely linked to poor prognosis. Given that hypopharyngeal and laryngeal SCCs are typically associated with tobacco and alcohol use, FGFR1 alterations are key driving factors in the carcinogenesis of these tobacco- and alcohol-related tumors [26].

FGFR1 amplification is a cause of tumor resistance. Related studies have shown that, during adaptation to EGFR-TKIs (Gefitinib), certain NSCLC cell lines exhibit a significant increase in FGF2 and FGFR1 mRNA and protein levels. The upregulation of FGFR1 provides an autocrine growth loop for tumor cells, allowing them to continue growing and proliferating despite EGFR inhibition [27]. NSCLC models with FGFR1 gene amplification exhibit resistance to the EGFR inhibitor gefitinib. This implies that, in the context of EGFR mutations, FGFR1 amplification or overactivation can lead to resistance to EGFR-targeted therapies [28]. Lenvatinib (E7080) was the second drug to receive approval for the treatment of hepatocellular carcinoma (HCC). Research has demonstrated that the overexpression of FGFR1 can hyperactivate the AKT/mTOR and ERK signaling pathways, leading to significant resistance to Lenvatinib in HCC [29].

### 3.3. Fusions and/or Rearrangements

FGFR1 rearrangements are closely associated with gene fusions. These rearrangements typically involve structural alterations in the FGFR1 gene, such as translocations, insertions, inversions, or deletions, which may lead to the fusion of FGFR1 with other genes, resulting in fusion genes that encode chimeric proteins [30]. These fusion proteins often dimerize or oligomerize, leading to the constitutive activation of FGFR1 kinase, which subsequently triggers aberrant downstream signaling pathways, promoting uncontrolled cell proliferation and tumorigenesis [31].

FGFR fusions are classified into two types: Type I, which arises from chromosomal translocations in hematologic malignancies, and Type II, which results from chromosomal rearrangements in solid tumors. Both types of FGFR fusion proteins achieve ligand-independent dimerization and/or abnormal substrate recruitment through protein–protein interaction modules obtained from their fusion partners, thereby conferring oncogenic potential [32]. At least 16 different FGFR1 fusion partner genes have been identified, including ZMYM2, FGFR1OP, CNTRL, ERVK3-1, BCR, NUP98, FGFR1OP2, TRIM24, MYO18A, CPSF6, LRRFIP1, CUX1, TPR, RANBP2, SQSTM1, and TFG, with most FGFR1 fusions leading to constitutive FGFR1 activation [33].

Type I FGFR fusion proteins are non-receptor FGFR fusion proteins with the N-terminus replaced by a fusion partner [34]. Several types are associated with FGFR1, including BCR-FGFR1, CNTRL-FGFR1, CUX1-FGFR1, FGFR1OP-FGFR1, FGFR1OP2-FGFR1, LRRFIP1-FGFR1, MYO18A-FGFR1, RANBP2-FGFR1, TPR-FGFR1, TRIM24-FGFR1, ZMYM2-FGFR1, CEP43-FGFR1, and ETV6-FGFR3 [32,34]. Type I FGFR fusion proteins acquire oncogenic potential by altering subcellular localization due to the loss of the extracellular and transmembrane domains of wild-type FGFR [32]. Specifically, the fusion gene is located at the 3′ end of the FGFR1 gene, where the FGFR1 tyrosine kinase domain fuses with the N-terminal oligomerization domain of the partner protein. The N-terminal oligomerization domain of the partner protein generates a fusion protein that cannot bind to FGF ligands, leading to a conformational change in the FGFR1 tyrosine kinase domain [35]. The resulting FGFR1 fusion protein induces a constitutive activation state, activating downstream cell signaling pathways, including PI3K-AKT, RAS/MAPK, STAT, and PLCγ/PKC, thereby transmitting aberrant signals [36,37]. For example, FGFR1 fuses with the Breakpoint Cluster Region (BCR) gene [38], producing a BCR-FGFR1 fusion protein that retains the coiled-coil oligomerization domain, serine/threonine kinase domain, and part of the RhoGEF domain of BCR. This fusion leads to the constitutive activation of FGFR1; BCR-FGFR1 activates the ERK/MAPK and JAK/STAT pathways and exhibits transformative activity in NIH3T3 cells, promoting tumor development [39]. BCR and ZMYM2 are the most common partner genes [40]. The proline-rich region in the protein oligomerization domain of ZMYM2 (previously known as ZNF198) and the FGFR1 tyrosine kinase domain maintain their conservation in the fusion protein [41]. The proline-rich domain of ZNF198 acts as a novel oligomerization domain that promotes constitutive activity of the ZNF198-FGFR1 tyrosine kinase. Aberrant oligomerization of ZMYM2-FGFR1 results in the constitutive activation of its tyrosine kinase. The ZMYM2-FGFR1 fusion protein contains the zinc finger domain of ZMYM2, mediating the constitutive activation of FGFR1 and causing sustained activation of the Notch pathway, which is closely related to the development of T-cell acute lymphoblastic leukemia (T-ALL) [30]. Additionally, the novel proline-rich oligomerization domain of ZNF198 is crucial for self-association, kinase activation, and cellular transformation, making it a potential target for drug development [42].

Type II FGFR fusion proteins are receptor-type FGFR fusion proteins with the C-terminus or N-terminus replaced by a fusion partner. [34]. The types associated with FGFR1 include FN1–FGFR1 and FGFR1-TACC1 [32,34]. Type II FGFR fusion proteins lose the tyrosine residue that binds to PLC-γ, such as Y766 in FGFR1, due to changes at the C-terminus [43]. The structure of the FGFR-TACC chromosomal rearrangement is a recurrent inversion. A distinctive feature of TACC proteins is the coiled-coil domain at the C-terminus, known as the TACC domain which, along with its dimerization ability, promotes fusion protein dimerization, autophosphorylation, and FGFR tyrosine kinase activation [44]. Aberrant expression of FGFR3-TACC3 triggers the activation of extracellular signal-regulated kinase (ERK/MAPK/MAPK) in specific cell types, while it may also activate the PI3K/Akt and signal transducer and activator of transcription 3 (STAT3) signaling pathways [45].

## 4. FGFR1-Targeted Therapeutics and Tumor Resistance

### 4.1. Kinase Domain-Targeting Drugs of FGFR1

**Figure 3:**
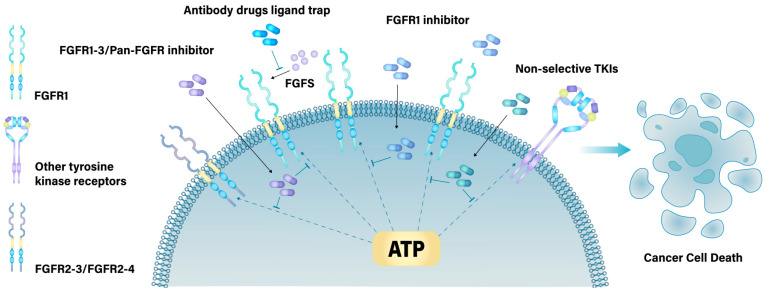
Drugs that target FGFR1. FGFR1-related drugs can be categorized into two main classes: intracellular domain-targeting drugs and extracellular domain-targeting drugs. Intracellular domain-targeting drugs bind to FGFR1 either covalently or non-covalently, competing with ATP for the binding site and inhibiting FGFR1 activity. Extracellular domain-targeting drugs primarily prevent FGFR1 from interacting with FGFs or cofactors, leading to the inactivation of FGFR1. Both classes of targeted therapies disrupt the associated signaling pathways through FGFR1 inhibition, ultimately resulting in tumor cell death.

Small-molecule inhibitors and biologic agents to target FGFRs have been developed as targeted therapies for patients with cancers that harbor FGFR alterations. Current small molecule drugs are primarily categorized into tyrosine-kinase inhibitors (TKIs) targeting specific kinases and small-molecule FGF receptor (FGFR) kinase alterations. The efficacy of these drugs varies depending on the specific FGFR1 alterations, emphasizing the importance of selecting medications based on the FGFR1 alterations profile for optimal treatment outcomes (Figure 3).

#### 4.1.1. Non-Selective Tyrosine-Kinase Inhibitors (TKIs)

Non-selective inhibitors are characterized by their ability to inhibit FGFR as well as other kinases, such as vascular endothelial growth factor receptor (VEGFR), platelet-derived growth factor receptor (PDGFR), and fetal liver tyrosine kinase receptor (FLT). FGFR1 is a primary target site for most Nonselective Tyrosine-Kinase Inhibitors (TKIs), and some nonselective TKIs have shown efficacy in tumors with FGFR1 aberrations. Many nonselective tyrosine-kinase inhibitors have been shown to be effective against tumors with FGFR1 aberrations, as shown in Table 1.

Dovitinib (TKI258) and Lucitanib are both orally administered TKIs capable of inhibiting FGF receptor, VEGF receptor (VEGFR), and platelet-derived growth factor receptor tyrosine kinases. Research indicates that both Dovitinib and Lucitanib exhibit significant inhibitory effects specifically in breast cancer patients with FGFR1 amplification, while sparing normal cells from adverse effects [46,47]. In mouse studies, Ponatinib exhibited significant inhibitory activity against fusion kinases, such as ZMYM2-FGFR1, BCR-FGFR1, and CEP110-FGFR1, subsequently inhibiting the phosphorylation and activation of downstream effectors, including PLCγ, Stat5, and Src [48]. Human studies have shown that Ponatinib significantly inhibits the activity of the fusion gene BCR-FGFR1, reducing its expression and thereby inhibiting aberrant signal transduction caused by the BCR-FGFR1 fusion gene. This suggests potential efficacy in treating trilineage mixed-phenotype acute leukemia [35].

A Phase 0/I trial on pancreatic cancer with FGFR1 amplification demonstrated that combined treatment with Nintedanib (200 mg/bid) and Letrozole resulted in a 55% average increase in plasma FGF23 levels, indicating effective FGFR1 inhibition [36]. Nintedanib may also have effects on FGFR1 point mutations [37]. Preclinical studies of Pazopanib have shown its inhibitory effects on FGFR1 fusion gene-positive tumors in both in vitro cell cultures and in vivo animal models. Patients with FGFR1_TACC1 treated with Pazopanib and Topotecan showed tumor size reduction and decreased leptomeningeal enhancement on MRI, indicating disease stabilization [45]. Pazopanib has also shown efficacy in FGFR1-amplified breast cancer and uterine carcinosarcoma [49,50].

Dasatinib has demonstrated inhibitory activity in FGFR1-rearranged diseases, such as EMS with CEP110-FGFR1 rearrangements, by blocking SRC kinases and has shown efficacy in Myeloproliferative Neoplasia [12]. Anlotinib inhibits the growth of EGFR-TKI-resistant NSCLC cells without the T790M mutation in vitro and in a mouse xenograft model by targeting overexpressed FGFR1 [51]. GZD824 (also known as HQP1351) is a third-generation ABL inhibitor that has completed Phase II clinical trials in China for chronic myeloid leukemia (CML) patients resistant to Abl mutation. The FGFR1-V561F/M mutant (gatekeeper mutation) exhibits resistance to several other FGFR inhibitors, such as erdafitinib, pemigatinib, BGJ398, and TAS120. However, GZD824 binds to FGFR1 in a manner that does not rely on direct hydrogen bonding or spatial arrangement with the gatekeeper residue, thereby bypassing the resistance caused by these mutations and effectively inhibiting the target. Studies have shown that GZD824 has a significant inhibitory effect on FGFR1 mutations or fusions, specifically in 8p11 myeloproliferative syndrome (EMS) patients [52,53]. Tissue experiments indicate that Sulfatinib exerts antitumor effects in osteosarcoma cells by inhibiting bFGF-induced epithelial–mesenchymal transition (EMT) through blocking FGFR1 phosphorylation [11].

However, studies have indicated that the inhibitory effects of TKIs on FGFR1 vary among different tumor types. A Phase II clinical trial evaluating Dovitinib in advanced squamous non-small cell lung cancer (NSCLC) patients with FGFR1 amplification did not observe a clear correlation between FGFR1 amplification levels and partial response (PR) rates. This suggests that FGFR1 amplification may not be an ideal target for Dovitinib [29].

The primary goal of using non-selective TKIs to treat FGFR1-driven tumors is to inhibit FGFR1 but, due to their limited selectivity, they also often interact with other kinases. This unintended inhibition can trigger off-target effects, resulting in various adverse reactions. These side effects may include gastrointestinal disturbances, fatigue, and other complications that arise from disrupting normal kinase functions beyond the FGFR1 pathway. For instance, inhibitors such as dovitinib and lucitanib target FGFR1, VEGFRs, and PDGFRs. This multi-kinase inhibition can lead to hypertension and other vascular complications due to the disruption of angiogenesis pathways [54]. Additionally, certain drugs inhibit kinases associated with cardiac function, thereby increasing the risk of arrhythmias or heart failure. In the liver, these inhibitors interfere with multiple key kinases involved in metabolic processes, causing elevated liver enzyme levels or even hepatotoxicity [55]. Gastrointestinal side effects, such as diarrhea, nausea, and vomiting, are also common and are linked to the inhibition of kinases involved in maintaining gastrointestinal mucosal integrity [56]. Therefore, developing more selective FGFR1 inhibitors is crucial to minimize these off-target effects, enhance therapeutic efficacy, and improve patient outcomes.

**Table 1:** Nonselective Tyrosine-Kinase Inhibitors with Efficacy Against FGFR1 Aberrations.

Drug	Type	Testee (Phase)	Tumor	Types of FGFR1 Aberration	Refs
Anlotinib	TKI	Mouse	Non-small cell lung cancer	Overexpression	[51]
Dasatinib	TKI	Human	Myeloproliferative neoplasia	Rearrangement	[12]
Dovitinib (TKI258)	TKI	Human (I)	Breast cancer	Amplification	[47]
Lucitanib (E-3810)	TKI	Human (I/II)	Breast cancer	Amplification	[46]
Nintedanib (BIBF 1120)	TKI	Human (0/I) Human	Breast cancer Non-small cell lung cancer	Amplification Point mutations	[36] [37]
Olverembatinib (GZD824/ HQP1351)	TKI	Human	Myeloproliferative syndrome	Fusion	[53]
Pazopanib (GW786034)	TKI	Human	Low-grade neuroepithelial tumor Uterine carcinosarcoma	Fusion Amplification	[45] [46]
Ponatinib (AP24534)	TKI	Mouse Human	Myeloid and lymphoid malignancies Acute leukemia	Fusion Fusion	[48] [35]
Sulfatinib (HMPL-012)	TKI	Tissue	Osteosarcoma	Amplification	[11]

#### 4.1.2. Selective Tyrosine-Kinase Inhibitors (TKIs)

FGFR-selective TKIs can be categorized into three classes: the first class consists of pan-FGFR inhibitors, which effectively inhibit FGFR1-4; the second class includes FGFR1/2/3 inhibitors, which effectively inhibit FGFR1-3 while exhibiting minimal inhibitory effects on FGFR4; the third class comprises FGFR1-selective inhibitors, which demonstrate high selectivity for FGFR1. Selective FGFR inhibitors are summarized in Table 2.

##### Pan-FGFR Inhibitors

Erdafitinib is an orally administered selective pan-FGFR tyrosine kinase inhibitor. A phase II clinical study demonstrated that Erdafitinib has significant efficacy against solid tumors with FGFR1 mutations (Lys656Glu) and FGFR1 fusions [57]. Futibatinib, a novel, potent, selective, and irreversible inhibitor of FGFR1-4, has shown a broad spectrum of antitumor activity in cell lines and xenograft models [58]. In a patient with a PCM1-FGFR1 gene rearrangement-driven myeloproliferative neoplasm, treatment with Futibatinib led to sustained complete hematologic and cytogenetic remission. This represents the first reported case of durable remission achieved with Futibatinib in an FGFR1-driven myeloproliferative neoplasm [59]. Rogaratinib is an oral pan-fibroblast growth factor receptor (FGFR1-4) inhibitor. Preliminary analysis from the FORT-1 study demonstrated that Rogaratinib exhibits an objective response rate and overall survival comparable to chemotherapy in patients with FGFR1/3 mRNA-positive tumors, with a manageable safety profile [60]. FIIN-2 and FIIN-3 are novel covalent FGFR inhibitors that target the conserved cysteine residue Cys475 in the P-loop (phosphate-binding loop) of the FGFR1 kinase domain via their acrylamide groups. The acrylamide group forms a covalent bond with Cys475 in the P-loop, resulting in a conformational shift of the DFG (Asp-Phe-Gly) motif of FGFR1 from its normal DFG-in conformation to a DFG-out conformation. This covalent binding inhibits FGFR1 activity, thereby blocking the PI3K/AKT/mTOR and RAS/MAPK pathways and suppressing the survival and proliferation of FGFR1-amplified and mutated non-small-cell lung cancer cells [61].

Some pan-FGFR1 inhibitors have not yet undergone specific experiments targeting FGFR1 but may serve as potential FGFR1 inhibitors in the future. KIN-3248 is a selective, irreversible, orally bioavailable small molecule inhibitor designed to target FGFR1-4, including FGFR1. A phase I clinical trial evaluated the safety and preliminary efficacy of KIN-3248 in patients with advanced solid tumors harboring FGFR2 and FGFR3 alterations. The study showed that KIN-3248 was generally well tolerated and exhibited favorable pharmacokinetic properties. Some patients showed partial responses to the treatment, indicating its potential efficacy against tumors driven by FGFR alterations, suggesting its potential application as an FGFR1 inhibitor [62]. PRN1371 has already been shown to be effective against tumors with FGFR3 fusions and FGFR2 amplification, making it a potential highly selective drug for FGFR1 variants [63]. Compound 10e is a dual FGFR/HDAC (fibroblast growth factor receptor/histone deacetylase) inhibitor designed to target FGFR1. It competes for the ATP-binding pocket of FGFR1 and exhibits a potent inhibitory effect, with an IC50 value of 0.18 nM against FGFR1. Compound 10e has demonstrated the ability to induce G0/G1 phase arrest and apoptosis in the SNU-16 cell line, exerting effects across various tumor cell types. However, no studies have yet confirmed the efficacy of compound 10e against specific FGFR1 variants [64]. PRN1371 is an irreversible covalent fibroblast growth factor receptor (FGFR1-4) inhibitor that targets the Cys488 site in FGFR1 through its acrylamide group in the acrylamido-piperazine moiety. Cys488 is located within a flexible glycine-rich loop in the ATP-binding pocket of FGFR1. This covalent binding mechanism grants PRN1371 high affinity and selectivity for FGFR1-4 [65].

##### FGFR1/2/3 Inhibitors

Some FGFR1/2/3 inhibitors have demonstrated stronger inhibitory effects on FGFR1. Infigratinib (BGJ398) is an effective ATP-competitive inhibitor that primarily targets FGFR1/2/3. This efficacy is evidenced by the high incidence of hyperphosphatemia, indicating a disruption of FGFR-related phosphate homeostasis. Although Infigratinib is considered as a Pan-FGFR inhibitor, it is more accurately classified as an FGFR1/2/3 inhibitor due to its higher IC50 for FGFR4 [66]. Studies have shown that Infigratinib is effective in treating FGFR1-amplified squamous non-small cell lung cancer (sqNSCLC), gliomas with FGFR1 mutations, and urothelial carcinoma with FGFR1 overexpression [66,67,68]. ARQ 087 inhibited the autophosphorylation of FGFR1 and FGFR2 in a dose-dependent manner, suggesting that, in addition to inhibiting the active (phosphorylated) form of the kinase, ARQ 087 binds to the unphosphorylated or inactive form of the kinase and delays its activation. In one patient with adrenocortical carcinoma whose tumor exhibited FGFR1 amplification, durable stable disease was observed following treatment with ARQ 087 [69].

Some drugs also hold significant potential for the treatment of FGFR1-related tumors. The pyrazole–benzimidazole derivative CPL304110 (compound 56q) is a potent and selective fibroblast growth factor receptor (FGFR) inhibitor. The benzimidazole moiety forms a hydrogen bond with the carbonyl group of Ala564 through its nitrogen atom, while the pyrazole moiety forms a hydrogen bond with the carbonyl group of Glu562 through its nitrogen atom. Additionally, the 3,5-dimethoxyphenyl group forms a hydrogen bond with Asp641 in the active site of FGFR1, contributing to increased binding affinity of the compound to the receptor. These interactions result in the formation of a stable complex that competitively binds to the ATP-binding domain of FGFR, thereby inhibiting FGFR activity [70]. Debio 1347 (also known as CH5183284) is an oral selective fibroblast growth factor receptor (FGFR) inhibitor that binds to Phe642 and Met535 in a back pocket of FGFR1. By binding to these residues, Debio 1347 effectively blocks the ATP-binding site of FGFR1, preventing ATP from binding and thereby inhibiting FGFR1 activity [71]. Studies have shown that Debio 1347 may be effective against tumors with FGFR1 amplification [72], but it does not exhibit significant efficacy in patients with FGFR1 fusions.

##### FGFR1 Selective Inhibitors

Small molecule inhibitors may exhibit limited discrimination among FGFR1, 2, 3, and 4 due to the high homology of their kinase domains. However, advancements in structural insights have led to the development of FGFR1 inhibitors with enhanced target specificity.

FGFR1 inhibitors have demonstrated efficacy against tumors with FGFR1 amplification. UPR1376 is a novel irreversible FGFR1 inhibitor that inactivates the enzyme by forming a covalent bond with the active site. UPR1376 contains a chloroacetamide functional group, which serves as a warhead that covalently binds to cysteine (Cys488) in the active site of FGFR1, irreversibly inhibiting FGFR1 phosphorylation and activation. By inhibiting FGFR1 activation, UPR1376 blocks ERK1/2 phosphorylation, thereby affecting cell proliferation and survival. Concurrently, it impacts AKT phosphorylation, inhibiting mTOR and p70S6K activation, thus affecting tumor cell growth, metabolism, and survival [73]. PD 173074 is a selective inhibitor that targets the ATP-binding pocket of FGFR1. Studies have shown that PD 173074 significantly reduces the phosphorylation levels of the MAPK pathway in non-small cell lung cancer (NSCLC) with FGFR1 amplification, subsequently reducing ERK phosphorylation, a member of the MAPK family, affecting tumor cell proliferation [73].

Although there is currently insufficient evidence to suggest that some FGFR1 inhibitors are effective against tumors with specific FGFR1 mutations, their high selectivity makes these drugs promising for tumors harboring FGFR1 mutations. CYY292 is a newly designed small-molecule FGFR1 inhibitor with strong affinity. CYY292 forms hydrogen bonds with the ALA564 and GLU562 residues of FGFR1 and, upon binding, acts on the Ser777 residue and phosphorylates it. This negative feedback mechanism reduces the phosphorylation of Y653 and Y654, thereby inhibiting the FGFR1/AKT/Snail signaling pathway to suppress the proliferation and invasion of glioblastoma (GBM) cells. CYY292 can penetrate the blood–brain barrier and has shown significant antitumor effects in GBM [74]. Compound 15c is a novel dual-target inhibitor that targets both EGFRL858R/T790M and FGFR1, inhibiting the kinase activity of both. The resistance of NSCLC to EGFR-TKIs is primarily due to FGFR1 overexpression leading to bypass activation. The dual-target inhibition by compound 15c effectively overcomes NSCLC cell resistance to EGFR-TKIs [75].

**Table 2:** Selective Tyrosine-Kinase Inhibitors with Efficacy Against FGFR1 Aberrations.

Drug	Testee (Phase)	Type	Tumor	Types of FGFR1 Aberration	Refs
Erdafitinib (JNJ-42756493)	Human (II)	Pan-FGFR inhibitors	Glioma endometrial cancer Pancreatic cancer High-grade glioma	Point mutations Fusion Fusion	[57] [57] [57]
Futibatinib (TAS-120)	Human	Pan-FGFR inhibitors	Myeloid neoplasm	Rearrangement	[59]
Rogaratinib (BAY-1163877)	Human (II/III)	Pan-FGFR inhibitors	Metastatic urothelial carcinoma	Overexpressing	[60]
FIIN-2	Cell	Pan-FGFR inhibitors	Non-small-cell lung cancer	Point mutations Amplifications	[61] [61]
FIIN-3	Cell	Pan-FGFR inhibitors	Non-small-cell lung cancer	Point mutations Amplifications	[61] [61]
Pemigatinib	Human (II)	FGFR1/2/3 inhibitors	Multiple lymphomas (MLNs)	Rearrangements	[10]
Infigratinib (BGJ398)	Cell Human (II)	FGFR1/2/3 inhibitors	Urothelial carcinoma None-small-cell lung cancer Gliomas	Overexpression Amplifications Point mutations	[66] [67] [68]
Derazantinib (ARQ087)	Human (I)	FGFR1-3 inhibitor	Adrenocortical carcinoma	Amplification	[69]
UPR1376	Cell	FGFR1 inhibitor	None-small-cell lung cancer	Amplifications	[73]
CYY292	Cell	FGFR1 inhibitor	Glioblastoma	Unknown	[74]
Compound15c	Mouse	EGFR^L858R/T790M^ and FGFR1	Non-small-cell lung cancer	Amplification	[75]
PD 173074	Cell	FGFR1 inhibitor	Squamous cell lung cancer	Amplifications	[76]

### 4.2. Extracellular Domain Targeting Drugs of FGFR1

Other therapeutic strategies, including FGFR1-specific monoclonal antibodies and ligand traps, have been developed to target the immunoglobulin-like domains of FGFR1. These approaches aim to inhibit the interaction between FGFR1 and its ligands, such as FGF, thereby exerting their therapeutic effects. These modalities represent emerging investigational approaches in the field (Figure 3).

#### 4.2.1. FGFR1 Antagonists and Antibody Drugs

Employing monoclonal antibodies (mAbs) to target molecules on the surface of cancer cells represents a cutting-edge therapeutic approach. This strategy has seen success with mAbs designed to bind to surface molecules associated with cancer, including the epidermal growth factor receptor (EGFR), human epidermal growth factor receptor 2 (HER2), and CD20. Antibody drugs targeting FGFR1 can bind to the extracellular domain of FGFR1, competing with FGFs for the binding site and thus inhibiting FGFR1 activity to achieve antitumor effects [77]. Sasaki et al. developed a novel monoclonal antibody (mAb) against FGFR1, named A2C9-1, which specifically binds to FGFR1 and blocks the interaction between FGF and FGFR1. When used in combination with interferon (IFN-α/β), this antibody significantly reduced HCC cell proliferation [78].There are also some treatment options that are based primarily on conjugation to the drug. Yang et al. screened a murine monoclonal antibody, mAbE12, which interferes with the interaction between bFGF and FGFR1, demonstrating the ability to inhibit the proliferation and tumor growth of lung adenocarcinoma (LLC) cells in both in vitro and in vivo experiments [79]. Borek et al. used phage display technology to screen a high-affinity scFv antibody fragment with a low nanomolar dissociation constant for FGFR1, indicating extremely high binding affinity. By conjugating this antibody with the cytotoxin vcMMAE (maleimidocaproyl-Val-Cit-PABC-monomethyl auristatin E), the antibody–drug conjugate exhibited specific cytotoxic effects on lung cancer cells overexpressing FGFR1 [80]. Although clinical reports on FGFR1-targeting antibody drugs are currently limited, several FGFR-targeting antibodies have already entered clinical trials, including LY3076226 [81] and BAY 1187982 [82]. Clinical results indicate that these antibodies exhibit a certain efficacy against solid tumors. However, patients have experienced varying degrees of side effects, such as anemia, elevated aspartate aminotransferase (AST), proteinuria, and thrombocytopenia. These adverse effects may be attributed to the inhibition of multiple signaling pathways. Therefore, enhancing the selectivity of these treatments and reducing immune responses are both primary objectives for future development.

Some protein antagonists can specifically bind to the extracellular domain of FGFR1, blocking FGFR1 dimerization and ligand-mediated activation, thereby inhibiting downstream signaling pathways. M6123 is a novel IgG-like monovalent FGFR1-specific binder that exhibits enhanced Antibody-Dependent Cellular Cytotoxicity (ADCC) effects. The allosteric binding of M6123 stabilizes a conformation that inhibits the ability of the receptor to dimerize. Without the proper dimerization, FGFR1 cannot undergo the activation necessary for downstream signaling. It has been shown to significantly inhibit tumor growth in FGFR1-dependent human xenograft models without causing weight loss in tumor-bearing mice. Reports indicate that patients with non-small cell lung cancer (NSCLC) exhibiting FGFR1 gene amplification and FGFR1 protein overexpression demonstrate higher sensitivity to M6123 [83]. On the other hand, P48 is a non-linear short peptide antagonist targeting the extracellular immunoglobulin-like domain of FGFR1. Rather than competing with the FGF ligand for binding to the extracellular domain, it prevents receptor activation by inhibiting dimerization after FGF binding. It forms hydrogen bonds with residues Q284 and D320 in the extracellular domain of FGFR1, thereby blocking specific signaling pathways of FGFR1, inhibiting cell proliferation and invasion. P48 has shown significant antitumor activity in in vitro experiments with cell lines such as cervical cancer (Hela229), melanoma (B16-F10), lung cancer (NCI-H460), and gastric cancer (SGC-7901, MGC-803), as well as in xenograft tumor models of gastric cancer [84]. Research into peptide inhibitors targeting FGFR1 is still in its early stages, with much of the work remaining at the preclinical level. One significant challenge identified so far is the rapid degradation of these peptide inhibitors within the body, which leads to a very short plasma half-life [85]. Future studies are expected to focus on improving the stability of these peptides, aiming to enhance their half-life and therapeutic effectiveness, particularly in cancer treatment.

Antibody drugs can not only hinder the binding of FGFs but also obstruct the binding of heparin to FGFR1, preventing complex formation and thus blocking the activation of FGFR1. IMB-R1 antiserum targets the heparin-binding domain (HBD) of FGFR1, preventing heparin/heparan sulfate (HS) from binding to FGFR1. This interference disrupts the formation of the FGF/FGFR1 complex and the activation of the FGF/FGFR1 signaling axis. In vitro experiments have shown that IMB-R1 significantly inhibits the growth of cancer cells’ overexpression of FGFR1 and induces apoptosis. Compared to known FGFR1 inhibitors, IMB-R1 demonstrates stronger antitumor effects [86].

Antibody drugs can also obstruct the binding of other signaling molecules to FGFR1. Gremlin1 promotes tumor cell proliferation and resistance to androgen deprivation therapy (ADT) by binding to fibroblast growth factor receptor 1 (FGFR1) and activating downstream mitogen-activated protein kinase (MAPK) signaling pathways. Using a monoclonal antibody against Gremlin1 can inhibit the activation of FGFR1, thereby suppressing tumor cell proliferation and tumor growth [87].

#### 4.2.2. FGF Ligand Traps

A ligand trap is a molecular tool used to capture and neutralize specific growth factors or signaling molecules, thereby preventing them from binding to cell surface receptors and activating downstream signaling pathways, ultimately inhibiting tumor progression. GSK3052230 (also known as FP-1039) is a soluble fusion protein targeting fibroblast growth factor receptor 1 (FGFR1). This molecule binds to the extracellular domain of the FGFR1c isoform and is fused with the Fc domain of IgG1, creating a trap capable of capturing FGF ligands with high affinity for mitogenic FGFs. In an open-label phase Ib study, 47% of patients in the first-line treatment group (Arm A) achieved confirmed partial responses (PR), with no significant adverse effects observed, suggesting that ligand traps hold promise as a future therapeutic approach [88].

### 4.3. FGFR1-Targeted Therapy Resistance

Preclinical data and corresponding clinical observations suggest that the activation of bypass signaling pathways and the occurrence of secondary FGFR alterations are significant factors contributing to resistance against FGFR-targeted therapies [89].

#### 4.3.1. Occurrence of Secondary FGFR1 Alterations

Secondary mutations tend to arise predominantly within the tyrosine kinase domain, particularly affecting the αC helix, αC–β4 loop, β4 and β5 strands, kinase hinge, αE helix, and A loop. These mutations disrupt the accessibility of FGFR inhibitors to their respective drug-binding clefts [90,91,92].

Resistance mutations in FGFR1 often occur at so-called “gatekeeper” sites, such as V561M and V564F, which are point mutations that can occur on the gatekeeper residues within the tyrosine kinase domain drug-binding pocket [52]. These mutations can either be recurrent or become predominant during targeted therapy, and they confer resistance by interfering with the receptor’s affinity for targeted drugs. The amino acid substitutions on the gatekeeper residues alter the pattern of drug-FGFR1 interactions; such mutations limit the ability of FGFR1 inhibitors to enter the drug-binding pocket, resulting in the mutated FGFR1 protein remaining in an active state. Consequently, gatekeeper mutations confer resistance to FGFR1 inhibitors [93,94].Studies have shown that FGFR1 resistance to Erdafitinib, Futibatinib, Infigratinib, and Pemigatinib is caused by point mutations on the gatekeeper residues V561M and V564F [52,95].

To ensure the successful translation of preclinical studies and related experimental research into clinical application phases, the effectiveness of fibroblast growth factor receptor (FGFR) inhibitors against tumors carrying secondary gatekeeper mutations needs to be assessed in patient populations with complex genetic alterations, including, but not limited to, the coexistence of primary mutations or fusion genes with secondary mutations. To further enhance the efficacy of FGFR1-targeted therapy in cancer patients, it is particularly crucial to develop FGFR1 kinase inhibitors that are active against multiple compound variations.

#### 4.3.2. Bypass Signaling

Resistance to FGFR1 inhibitors, which is mediated by the activation of identical or analogous downstream effectors through alternative signaling pathways, known as bypass signaling, can be classified as either intrinsic or acquired resistance [90]. Bypassing FGFR1 by activating other receptor tyrosine kinases (RTKs) and downstream RAS-RAF-ERK and PI3K-AKT-mTOR signaling cascades provides alternative pathways for resistance mechanisms to FGFR inhibitors. Epidermal growth factor receptor (EGFR) and MET are representative examples of RTKs that can compensate for FGFR signal inhibition, which may be achieved through genetic and non-genetic mechanisms. Acquired functional alterations in KRAS and PIK3CA, as well as loss-of-function changes in PTEN, may lead to aberrant activation of downstream signaling cascades, independent of RTK activation [90]. In summary, the activation of non-FGFR receptor tyrosine kinases (RTKs), including EGFR and MET, along with downstream components of the RAS/MAPK or PI3K/AKT signaling pathways, facilitates bypass signaling. This mechanism can lead to both intrinsic and acquired resistance to therapies targeting FGFR. Therefore, in the future, synergistic therapy based on individualized sequencing may be the preferred option to overcome bypass activation resistance.

## 5. Conclusions and Prospects

The efficacy of FGFR1-targeted therapies varies depending on the specific genetic alterations and the type of tumor. For example, clinical studies have demonstrated that Fexagratinib (AZD4547), an FGFR inhibitor, shows moderate anti-tumor activity in patients with advanced squamous cell lung cancer (SQCLC) harboring FGFR1 amplification, but its effectiveness in estrogen receptor-positive metastatic breast cancer is suboptimal, with patients experiencing significant adverse effects [96]. Similarly, Sunitinib has shown limited efficacy in patients with FGFR1-amplified metastatic breast cancer, with a substantial number of grade 3–4 adverse events or severe side effects reported [97]. These findings highlight the importance of tailoring FGFR1-targeted treatments according to the specific molecular characteristics of the tumor and the type of FGFR1 aberration present. The development of next-generation FGFR inhibitors with greater selectivity and potency against specific FGFR1 alterations holds promise for improving clinical outcomes. For instance, studies have reported that, in an effort to enhance drug targeting, researchers developed a selective FGFR1/2-targeting proteolysis-targeting chimera, BR-cpd7, which demonstrates significant isoform specificity for FGFR1/2. Importantly, BR-cpd7 exhibits almost no antiproliferative activity against cancer cells without FGFR aberrations, further substantiating its selectivity [98].

Despite the advancements in FGFR1-targeted therapies, several challenges remain. Current FGFR inhibitors, particularly non-selective tyrosine kinase inhibitors (TKIs), often result in off-target effects and dose-limiting toxicities, which restrict their clinical utility. Furthermore, the emergence of resistance, due to secondary mutations in the FGFR1 kinase domain, complicates treatment strategies. FGFR1-selective inhibitors, which specifically target FGFR1 without affecting other FGFR family members, offer a more targeted approach and have demonstrated encouraging preclinical and early clinical results in overcoming these challenges. These selective inhibitors, such as novel covalent inhibitors and monoclonal antibodies targeting extracellular domains, have shown potential in mitigating adverse effects and enhancing therapeutic efficacy.

The future development of FGFR1-targeted therapies should prioritize the discovery of more potent and highly selective inhibitors that can effectively target specific FGFR1 mutations or fusions while minimizing toxicity. The next generation of FGFR1 inhibitors should prioritize enhanced selectivity to minimize off-target effects, focusing on developing highly specific inhibitors that reduce interference with other kinases. Allosteric modulation and biomarker-driven strategies are promising directions to improve targeting precision and treatment outcomes, respectively. Additionally, patient stratification using companion diagnostics can optimize efficacy by identifying individuals most likely to benefit from these treatments.

To reduce toxicity and improve efficacy, novel delivery methods, such as targeted nanoparticles or antibody-drug conjugates, can ensure higher drug concentrations at tumor sites while sparing healthy tissue. Combining FGFR1 inhibitors with other therapies, such as immune checkpoint inhibitors, PROTAC-based drugs, targeted agents, or chemotherapy, could simultaneously address multiple pathways involved in cancer progression and resistance, enhancing therapeutic effects while reducing doses and associated toxicity. This approach is further supported by advancements in precision oncology, such as comprehensive genomic profiling, which enables the identification of patients most likely to benefit from FGFR1-targeted treatments. Moreover, a deeper understanding of the mechanisms of action of FGFR1-targeted drugs in patients with specific FGFR1 alterations will be crucial. Elucidating these mechanisms will optimize treatment strategies and improve outcomes for patients with FGFR1-driven tumors.

## Data Availability

All data generated or analyzed during this study are included in this published article.

## References

[1] Thein K.Z., Myat Y.M., Park B.S., Panigrahi K., Kummar S. (2024). Target-Driven Tissue-Agnostic Drug Approvals-A New Path of Drug Development. Cancers.

[2] Roskoski R. (2020). The role of fibroblast growth factor receptor (FGFR) protein-tyrosine kinase inhibitors in the treatment of cancers including those of the urinary bladder. Pharmacol. Res..

[3] Yang W., Sun Y. (2021). Promising Molecular Targets for the Targeted Therapy of Biliary Tract Cancers: An Overview. OncoTargets Ther..

[4] Goida J., Pilmane M. (2022). The Evaluation of FGFR1, FGFR2 and FOXO1 in Orofacial Cleft Tissue. Children.

[5] Soutto M., Zhang X., Bhat N., Chen Z., Zhu S., Maacha S., Genoula M., El-Gazzaz O., Peng D., Lu H. (2024). Fibroblast growth factor receptor-4 mediates activation of Nuclear Factor Erythroid 2-Related Factor-2 in gastric tumorigenesis. Redox Biol..

[6] Farrell B., Breeze A.L. (2018). Structure, activation and dysregulation of fibroblast growth factor receptor kinases: Perspectives for clinical targeting. Biochem. Soc. Trans..

[7] Dienstmann R., Rodon J., Prat A., Perez-Garcia J., Adamo B., Felip E., Cortes J., Iafrate A.J., Nuciforo P., Tabernero J. (2014). Genomic aberrations in the FGFR pathway: Opportunities for targeted therapies in solid tumors. Ann. Oncol. Off. J. Eur. Soc. Med. Oncol..

[8] Liu Q., Huang J., Yan W., Liu Z., Liu S., Fang W. (2023). FGFR families: Biological functions and therapeutic interventions in tumors. MedComm.

[9] Helsten T., Elkin S., Arthur E., Tomson B.N., Carter J., Kurzrock R. (2016). The FGFR Landscape in Cancer: Analysis of 4,853 Tumors by Next-Generation Sequencing. Clin. Cancer Res. Off. J. Am. Assoc. Cancer Res..

[10] Rodón J., Damian S., Furqan M., García-Donas J., Imai H., Italiano A., Spanggaard I., Ueno M., Yokota T., Veronese M.L. (2024). Pemigatinib in previously treated solid tumors with activating FGFR1-FGFR3 alterations: Phase 2 FIGHT-207 basket trial. Nat. Med..

[11] Liao S., Li J., Gao S., Han Y., Han X., Wu Y., Bi J., Xu M., Bi W. (2023). Sulfatinib, a novel multi-targeted tyrosine kinase inhibitor of FGFR1, CSF1R, and VEGFR1-3, suppresses osteosarcoma proliferation and invasion via dual role in tumor cells and tumor microenvironment. Front. Oncol..

[12] Wehrli M., Oppliger Leibundgut E., Gattiker H.H., Manz M.G., Müller A.M., Goede J.S. (2017). Response to Tyrosine Kinase Inhibitors in Myeloproliferative Neoplasia with 8p11 Translocation and CEP110-FGFR1 Rearrangement. Oncol..

[13] Ornitz D.M., Itoh N. (2015). The Fibroblast Growth Factor signaling pathway. Wiley Interdiscip. Rev. Dev. Biol..

[14] Thisse B., Thisse C. (2005). Functions and regulations of fibroblast growth factor signaling during embryonic development. Dev. Biol..

[15] Wu A.L., Kolumam G., Stawicki S., Chen Y., Li J., Zavala-Solorio J., Phamluong K., Feng B., Li L., Marsters S. (2011). Amelioration of type 2 diabetes by antibody-mediated activation of fibroblast growth factor receptor 1. Sci. Transl. Med..

[16] Stamou M.I., Chiu C.J., Jadhav S.V., Lopes V.F., Salnikov K.B., Plummer L., Lippincott M.F., Lee H., Seminara S.B., Balasubramanian R. (2024). Defective FGFR1 Signaling Disrupts Glucose Regulation: Evidence From Humans With FGFR1 Mutations. J. Endocr. Soc..

[17] Eming S.A., Brachvogel B., Odorisio T., Koch M. (2007). Regulation of angiogenesis: Wound healing as a model. Prog. Histochem. Cytochem..

[18] Cimmino F., Montella A., Tirelli M., Avitabile M., Lasorsa V.A., Visconte F., Cantalupo S., Maiorino T., De Angelis B., Morini M. (2022). FGFR1 is a potential therapeutic target in neuroblastoma. Cancer Cell Int..

[19] Bennett J.T., Tan T.Y., Alcantara D., Tétrault M., Timms A.E., Jensen D., Collins S., Nowaczyk M.J.M., Lindhurst M.J., Christensen K.M. (2016). Mosaic Activating Mutations in FGFR1 Cause Encephalocraniocutaneous Lipomatosis. Am. J. Hum. Genet..

[20] Rivera B., Gayden T., Carrot-Zhang J., Nadaf J., Boshari T., Faury D., Zeinieh M., Blanc R., Burk D.L., Fahiminiya S. (2016). Germline and somatic FGFR1 abnormalities in dysembryoplastic neuroepithelial tumors. Acta Neuropathol..

[21] Domenichini M., Ravelli C., Corsini M., Codenotti S., Moreschi E., Gogna A., Capoferri D., Zizioli D., Bresciani R., Grillo E. (2023). The D647N mutation of FGFR1 induces ligand-independent receptor activation. Biochim. Et Biophys. Acta. Gen. Subj..

[22] Sohl C.D., Ryan M.R., Luo B., Frey K.M., Anderson K.S. (2015). Illuminating the molecular mechanisms of tyrosine kinase inhibitor resistance for the FGFR1 gatekeeper mutation: The Achilles’ heel of targeted therapy. ACS Chem. Biol..

[23] Hu Y., Ai L.S., Zhou L.Q. (2021). Prognostic value of FGFR1 expression and amplification in patients with HNSCC: A systematic review and meta-analysis. PLoS ONE.

[24] Bockorny B., Rusan M., Chen W., Liao R.G., Li Y., Piccioni F., Wang J., Tan L., Thorner A.R., Li T. (2018). RAS-MAPK Reactivation Facilitates Acquired Resistance in FGFR1-Amplified Lung Cancer and Underlies a Rationale for Upfront FGFR-MEK Blockade. Mol. Cancer Ther..

[25] Ipenburg N.A., Koole K., Liem K.S., van Kempen P.M., Koole R., van Diest P.J., van Es R.J., Willems S.M. (2016). Fibroblast Growth Factor Receptor Family Members as Prognostic Biomarkers in Head and Neck Squamous Cell Carcinoma: A Systematic Review. Target. Oncol..

[26] Kim E.K., Cho Y.A., Koh Y.W., Shin H.A., Cho B.C., Yoon S.O. (2020). Prognostic implications of Fibroblast growth factor receptor 1 (FGFR1) gene amplification and protein overexpression in hypopharyngeal and laryngeal squamous cell carcinoma. BMC Cancer.

[27] Ware K.E., Hinz T.K., Kleczko E., Singleton K.R., Marek L.A., Helfrich B.A., Cummings C.T., Graham D.K., Astling D., Tan A.C. (2013). A mechanism of resistance to gefitinib mediated by cellular reprogramming and the acquisition of an FGF2-FGFR1 autocrine growth loop. Oncogenesis.

[28] Zhang X.C., Zhang J., Li M., Huang X.S., Yang X.N., Zhong W.Z., Xie L., Zhang L., Zhou M., Gavine P. (2013). Establishment of patient-derived non-small cell lung cancer xenograft models with genetic aberrations within EGFR, KRAS and FGFR1: Useful tools for preclinical studies of targeted therapies. J. Transl. Med..

[29] Zhao Z., Song J., Zhang D., Wu F., Tu J., Ji J. (2021). Oxysophocarpine suppresses FGFR1-overexpressed hepatocellular carcinoma growth and sensitizes the therapeutic effect of lenvatinib. Life Sci..

[30] Mertens F., Johansson B., Fioretos T., Mitelman F. (2015). The emerging complexity of gene fusions in cancer. Nat. Rev. Cancer.

[31] Chen L., Zhang Y., Yin L., Cai B., Huang P., Li X., Liang G. (2021). Fibroblast growth factor receptor fusions in cancer: Opportunities and challenges. J. Exp. Clin. Cancer Res. CR.

[32] Katoh M. (2016). FGFR inhibitors: Effects on cancer cells, tumor microenvironment and whole-body homeostasis (Review). Int. J. Mol. Med..

[33] Zhang X., Wang F., Yan F., Huang D., Wang H., Gao B., Gao Y., Hou Z., Lou J., Li W. (2022). Identification of a novel HOOK3-FGFR1 fusion gene involved in activation of the NF-kappaB pathway. Cancer Cell Int..

[34] Katoh M., Loriot Y., Brandi G., Tavolari S., Wainberg Z.A., Katoh M. (2024). FGFR-targeted therapeutics: Clinical activity, mechanisms of resistance and new directions. Nat. Rev. Clin. Oncol..

[35] Khodadoust M.S., Luo B., Medeiros B.C., Johnson R.C., Ewalt M.D., Schalkwyk A.S., Bangs C.D., Cherry A.M., Arai S., Arber D.A. (2016). Clinical activity of ponatinib in a patient with FGFR1-rearranged mixed-phenotype acute leukemia. Leukemia.

[36] Quintela-Fandino M., Apala J.V., Malon D., Mouron S., Hornedo J., Gonzalez-Cortijo L., Colomer R., Guerra J. (2019). Nintedanib plus letrozole in early breast cancer: A phase 0/I pharmacodynamic, pharmacokinetic, and safety clinical trial of combined FGFR1 and aromatase inhibition. Breast Cancer Res. BCR.

[37] Auberle C., Gao F., Sloan M., Morgensztern D., Winkler L., Ward J.P., Devarakonda S., Rearden T.P., Govindan R., Waqar S.N. (2024). A pilot study of nintedanib in molecularly selected patients with advanced non-small cell lung cancer. J. Thorac. Dis..

[38] Chong Y., Liu Y., Lu S., Cai B., Qin H., Chang C.S., Ren M., Cowell J.K., Hu T. (2020). Critical individual roles of the BCR and FGFR1 kinase domains in BCR-FGFR1-driven stem cell leukemia/lymphoma syndrome. Int. J. Cancer.

[39] Peiris M.N., Meyer A.N., Nelson K.N., Bisom-Rapp E.W., Donoghue D.J. (2020). Oncogenic fusion protein BCR-FGFR1 requires the breakpoint cluster region-mediated oligomerization and chaperonin Hsp90 for activation. Haematologica.

[40] Jackson C.C., Medeiros L.J., Miranda R.N. (2010). 8p11 myeloproliferative syndrome: A review. Hum. Pathol..

[41] Wang Y., Wu X., Deng J., Yu H., Xu R., Zhu Z., Tu S., Hu Y. (2016). Diagnostic application of next-generation sequencing in ZMYM2-FGFR1 8p11 myeloproliferative syndrome: A case report. Cancer Biol. Ther..

[42] Xiao S., McCarthy J.G., Aster J.C., Fletcher J.A. (2000). ZNF198-FGFR1 transforming activity depends on a novel proline-rich ZNF198 oligomerization domain. Blood.

[43] Lu M., Wang K., Ji W., Yu Y., Li Z., Xia W., Lu S. (2022). FGFR1 promotes tumor immune evasion via YAP-mediated PD-L1 expression upregulation in lung squamous cell carcinoma. Cell. Immunol..

[44] Lasorella A., Sanson M., Iavarone A. (2017). FGFR-TACC gene fusions in human glioma. Neuro-Oncol..

[45] Parker B.C., Annala M.J., Cogdell D.E., Granberg K.J., Sun Y., Ji P., Li X., Gumin J., Zheng H., Hu L. (2013). The tumorigenic FGFR3-TACC3 gene fusion escapes miR-99a regulation in glioblastoma. J. Clin. Investig..

[46] Guffanti F., Chilà R., Bello E., Zucchetti M., Zangarini M., Ceriani L., Ferrari M., Lupi M., Jacquet-Bescond A., Burbridge M.F. (2017). In Vitro and In Vivo Activity of Lucitanib in FGFR1/2 Amplified or Mutated Cancer Models. Neoplasia.

[47] André F., Bachelot T., Campone M., Dalenc F., Perez-Garcia J.M., Hurvitz S.A., Turner N., Rugo H., Smith J.W., Deudon S. (2013). Targeting FGFR with dovitinib (TKI258): Preclinical and clinical data in breast cancer. Clin. Cancer Res. Off. J. Am. Assoc. Cancer Res..

[48] Ren M., Qin H., Ren R., Cowell J.K. (2013). Ponatinib suppresses the development of myeloid and lymphoid malignancies associated with FGFR1 abnormalities. Leukemia.

[49] Nelson A.T., Bendel A., Skrypek M., Patel S., Tabori U., McDonald W., Schultz K.A.P. (2022). Leptomeningeal Dissemination of Low-Grade Neuroepithelial Tumor with FGFR1_TACC1 Fusion with Clinical and Radiographic Response to Pazopanib and Topotecan. Pediatr. Neurosurg..

[50] Cheng F.T., Ou-Yang F., Lapke N., Tung K.C., Chen Y.K., Chou Y.Y., Chen S.J. (2017). Pazopanib Sensitivity in a Patient With Breast Cancer and FGFR1 Amplification. J. Natl. Compr. Cancer Netw. JNCCN.

[51] Lian Z., Du W., Zhang Y., Fu Y., Liu T., Wang A., Cai T., Zhu J., Zeng Y., Liu Z. (2020). Anlotinib can overcome acquired resistance to EGFR-TKIs via FGFR1 signaling in non-small cell lung cancer without harboring EGFR T790M mutation. Thorac. Cancer.

[52] Jiang K., Tang X., Guo J., He R., Chan S., Song X., Tu Z., Wang Y., Ren X., Ding K. (2021). GZD824 overcomes FGFR1-V561F/M mutant resistance in vitro and in vivo. Cancer Med..

[53] Yan Y., Qu S., Liu J., Li C., Yan X., Xu Z., Qin T., Jia Y., Pan L., Gao Q. (2023). Olverembatinib for myeloid/lymphoid neoplasm associated with eosinophilia and FGFR1 rearrangement. Leuk. Lymphoma.

[54] Angevin E., Lopez-Martin J.A., Lin C.C., Gschwend J.E., Harzstark A., Castellano D., Soria J.C., Sen P., Chang J., Shi M. (2013). Phase I study of dovitinib (TKI258), an oral FGFR, VEGFR, and PDGFR inhibitor, in advanced or metastatic renal cell carcinoma. Clin. Cancer Res. Off. J. Am. Assoc. Cancer Res..

[55] Shah R.R., Morganroth J., Shah D.R. (2013). Hepatotoxicity of tyrosine kinase inhibitors: Clinical and regulatory perspectives. Drug Saf..

[56] Llovet J.M., Ricci S., Mazzaferro V., Hilgard P., Gane E., Blanc J.F., de Oliveira A.C., Santoro A., Raoul J.L., Forner A. (2008). Sorafenib in advanced hepatocellular carcinoma. N. Engl. J. Med..

[57] Pant S., Schuler M., Iyer G., Witt O., Doi T., Qin S., Tabernero J., Reardon D.A., Massard C., Minchom A. (2023). Erdafitinib in patients with advanced solid tumours with FGFR alterations (RAGNAR): An international, single-arm, phase 2 study. Lancet. Oncol..

[58] Sootome H., Fujita H., Ito K., Ochiiwa H., Fujioka Y., Ito K., Miura A., Sagara T., Ito S., Ohsawa H. (2020). Futibatinib Is a Novel Irreversible FGFR 1-4 Inhibitor That Shows Selective Antitumor Activity against FGFR-Deregulated Tumors. Cancer Res..

[59] Kasbekar M., Nardi V., Dal Cin P., Brunner A.M., Burke M., Chen Y.B., Connolly C., Fathi A.T., Foster J., Macrae M. (2020). Targeted FGFR inhibition results in a durable remission in an FGFR1-driven myeloid neoplasm with eosinophilia. Blood Adv..

[60] Sternberg C.N., Petrylak D.P., Bellmunt J., Nishiyama H., Necchi A., Gurney H., Lee J.L., van der Heijden M.S., Rosenbaum E., Penel N. (2023). FORT-1: Phase II/III Study of Rogaratinib Versus Chemotherapy in Patients With Locally Advanced or Metastatic Urothelial Carcinoma Selected Based on FGFR1/3 mRNA Expression. J. Clin. Oncol. Off. J. Am. Soc. Clin. Oncol..

[61] Tan L., Wang J., Tanizaki J., Huang Z., Aref A.R., Rusan M., Zhu S.J., Zhang Y., Ercan D., Liao R.G. (2014). Development of covalent inhibitors that can overcome resistance to first-generation FGFR kinase inhibitors. Proc. Natl. Acad. Sci. USA.

[62] Garmezy B., Borad M.J., Bahleda R., Perez C.A., Chen L.T., Kato S., Oh D.Y., Severson P., Tam B.Y., Quah C.S. (2024). A Phase I Study of KIN-3248, an Irreversible Small-molecule Pan-FGFR Inhibitor, in Patients with Advanced FGFR2/3-driven Solid Tumors. Cancer Res. Commun..

[63] Venetsanakos E., Brameld K.A., Phan V.T., Verner E., Owens T.D., Xing Y., Tam D., LaStant J., Leung K., Karr D.E. (2017). The Irreversible Covalent Fibroblast Growth Factor Receptor Inhibitor PRN1371 Exhibits Sustained Inhibition of FGFR after Drug Clearance. Mol. Cancer Ther..

[64] Wan G., Feng Z., Zhang Q., Li X., Ran K., Feng H., Luo T., Zhou S., Su C., Wei W. (2022). Design and Synthesis of Fibroblast Growth Factor Receptor (FGFR) and Histone Deacetylase (HDAC) Dual Inhibitors for the Treatment of Cancer. J. Med. Chem..

[65] Brameld K.A., Owens T.D., Verner E., Venetsanakos E., Bradshaw J.M., Phan V.T., Tam D., Leung K., Shu J., LaStant J. (2017). Discovery of the Irreversible Covalent FGFR Inhibitor 8-(3-(4-Acryloylpiperazin-1-yl)propyl)-6-(2,6-dichloro-3,5-dimethoxyphenyl)-2-(methylamino)pyrido [2,3-d]pyrimidin-7(8H)-one (PRN1371) for the Treatment of Solid Tumors. J. Med. Chem..

[66] Kim S.H., Ryu H., Ock C.Y., Suh K.J., Lee J.Y., Kim J.W., Lee J.O., Kim J.W., Kim Y.J., Lee K.W. (2018). BGJ398, A Pan-FGFR Inhibitor, Overcomes Paclitaxel Resistance in Urothelial Carcinoma with FGFR1 Overexpression. Int. J. Mol. Sci..

[67] Nogova L., Sequist L.V., Perez Garcia J.M., Andre F., Delord J.P., Hidalgo M., Schellens J.H., Cassier P.A., Camidge D.R., Schuler M. (2017). Evaluation of BGJ398, a Fibroblast Growth Factor Receptor 1-3 Kinase Inhibitor, in Patients With Advanced Solid Tumors Harboring Genetic Alterations in Fibroblast Growth Factor Receptors: Results of a Global Phase I, Dose-Escalation and Dose-Expansion Study. J. Clin. Oncol. Off. J. Am. Soc. Clin. Oncol..

[68] Lassman A.B., Sepúlveda-Sánchez J.M., Cloughesy T.F., Gil-Gil M.J., Puduvalli V.K., Raizer J.J., De Vos F.Y.F., Wen P.Y., Butowski N.A., Clement P.M.J. (2022). Infigratinib in Patients with Recurrent Gliomas and FGFR Alterations: A Multicenter Phase II Study. Clin. Cancer Res. Off. J. Am. Assoc. Cancer Res..

[69] Papadopoulos K.P., El-Rayes B.F., Tolcher A.W., Patnaik A., Rasco D.W., Harvey R.D., LoRusso P.M., Sachdev J.C., Abbadessa G., Savage R.E. (2017). A Phase 1 study of ARQ 087, an oral pan-FGFR inhibitor in patients with advanced solid tumours. Br. J. Cancer.

[70] Yamani A., Zdżalik-Bielecka D., Lipner J., Stańczak A., Piórkowska N., Stańczak P.S., Olejkowska P., Hucz-Kalitowska J., Magdycz M., Dzwonek K. (2021). Discovery and optimization of novel pyrazole-benzimidazole CPL304110, as a potent and selective inhibitor of fibroblast growth factor receptors FGFR (1-3). Eur. J. Med. Chem..

[71] Nakanishi Y., Akiyama N., Tsukaguchi T., Fujii T., Sakata K., Sase H., Isobe T., Morikami K., Shindoh H., Mio T. (2014). The fibroblast growth factor receptor genetic status as a potential predictor of the sensitivity to CH5183284/Debio 1347, a novel selective FGFR inhibitor. Mol. Cancer Ther..

[72] Voss M.H., Hierro C., Heist R.S., Cleary J.M., Meric-Bernstam F., Tabernero J., Janku F., Gandhi L., Iafrate A.J., Borger D.R. (2019). A Phase I, Open-Label, Multicenter, Dose-escalation Study of the Oral Selective FGFR Inhibitor Debio 1347 in Patients with Advanced Solid Tumors Harboring FGFR Gene Alterations. Clin. Cancer Res. Off. J. Am. Assoc. Cancer Res..

[73] Fumarola C., Bozza N., Castelli R., Ferlenghi F., Marseglia G., Lodola A., Bonelli M., La Monica S., Cretella D., Alfieri R. (2019). Expanding the Arsenal of FGFR Inhibitors: A Novel Chloroacetamide Derivative as a New Irreversible Agent With Anti-proliferative Activity Against FGFR1-Amplified Lung Cancer Cell Lines. Front. Oncol..

[74] Bi Y., Zheng R., Hu J., Shi R., Shi J., Wang Y., Wang P., Jiang W., Kim G., Liu Z. (2024). A novel FGFR1 inhibitor CYY292 suppresses tumor progression, invasion, and metastasis of glioblastoma by inhibiting the Akt/GSK3β/snail signaling axis. Genes Dis..

[75] Chen G., Bao Y., Weng Q., Zhao Y., Lu X., Fu L., Chen L., Liu Z., Zhang X., Liang G. (2019). Compound 15c, a Novel Dual Inhibitor of EGFR(L858R/T790M) and FGFR1, Efficiently Overcomes Epidermal Growth Factor Receptor-Tyrosine Kinase Inhibitor Resistance of Non-Small-Cell Lung Cancers. Front. Pharmacol..

[76] Weiss J., Sos M.L., Seidel D., Peifer M., Zander T., Heuckmann J.M., Ullrich R.T., Menon R., Maier S., Soltermann A. (2010). Frequent and focal FGFR1 amplification associates with therapeutically tractable FGFR1 dependency in squamous cell lung cancer. Sci. Transl. Med..

[77] Pomerantz R.G., Grandis J.R. (2004). The epidermal growth factor receptor signaling network in head and neck carcinogenesis and implications for targeted therapy. Semin. Oncol..

[78] Sasaki S., Ishida T., Toyota M., Ota A., Suzuki H., Takaoka A., Yasui H., Yamamoto H., Takagi H., Maeda M. (2011). Interferon-α/β and anti-fibroblast growth factor receptor 1 monoclonal antibody suppress hepatic cancer cells in vitro and in vivo. PLoS ONE.

[79] Yang Y., Luo Z., Qin Y., Zhou Y., Gong L., Huang J., Wang H. (2017). Production of bFGF monoclonal antibody and its inhibition of metastasis in Lewis lung carcinoma. Mol. Med. Rep..

[80] Sokolowska-Wedzina A., Chodaczek G., Chudzian J., Borek A., Zakrzewska M., Otlewski J. (2017). High-Affinity Internalizing Human scFv-Fc Antibody for Targeting FGFR1-Overexpressing Lung Cancer. Mol. Cancer Res. MCR.

[81] Kollmannsberger C., Britten C.D., Olszanski A.J., Walker J.A., Zang W., Willard M.D., Radtke D.B., Farrington D.L., Bell-McGuinn K.M., Patnaik A. (2021). A phase 1 study of LY3076226, a fibroblast growth factor receptor 3 (FGFR3) antibody-drug conjugate, in patients with advanced or metastatic cancer. Investig. New Drugs.

[82] Kim S.B., Meric-Bernstam F., Kalyan A., Babich A., Liu R., Tanigawa T., Sommer A., Osada M., Reetz F., Laurent D. (2019). First-in-Human Phase I Study of Aprutumab Ixadotin, a Fibroblast Growth Factor Receptor 2 Antibody-Drug Conjugate (BAY 1187982) in Patients with Advanced Cancer. Target. Oncol..

[83] Yu P., Knippel A., Onidi M., Paoletti A., Vigna E., Hellmann J., Esdar C. (2020). A novel monovalent FGFR1 antagonist: Preclinical safety profiles in rodents and non-human primates. Toxicol. Appl. Pharmacol..

[84] Wu J., Chen L., Chen L., Fan L., Wang Z., Dong Z., Chen Q., Wei T., Cai Y., Li W. (2020). The discovery of potent and stable short peptide FGFR1 antagonist for cancer therapy. Eur. J. Pharm. Sci. Off. J. Eur. Fed. Pharm. Sci..

[85] Craik D.J., Fairlie D.P., Liras S., Price D. (2013). The future of peptide-based drugs. Chem. Biol. Drug Des..

[86] Ling L., Tan S.K., Goh T.H., Cheung E., Nurcombe V., van Wijnen A.J., Cool S.M. (2015). Targeting the heparin-binding domain of fibroblast growth factor receptor 1 as a potential cancer therapy. Mol. Cancer.

[87] Sena L.A., Brennen W.N., Isaacs J.T. (2022). There are gremlins in prostate cancer. Nat. Cancer.

[88] Morgensztern D., Karaseva N., Felip E., Delgado I., Burdaeva O., Dómine M., Lara P., Paik P.K., Lassen U., Orlov S. (2019). An open-label phase IB study to evaluate GSK3052230 in combination with paclitaxel and carboplatin, or docetaxel, in FGFR1-amplified non-small cell lung cancer. Lung Cancer.

[89] Camidge D.R., Pao W., Sequist L.V. (2014). Acquired resistance to TKIs in solid tumours: Learning from lung cancer. Nat. Rev. Clin. Oncol..

[90] Perera T.P.S., Jovcheva E., Mevellec L., Vialard J., De Lange D., Verhulst T., Paulussen C., Van De Ven K., King P., Freyne E. (2017). Discovery and Pharmacological Characterization of JNJ-42756493 (Erdafitinib), a Functionally Selective Small-Molecule FGFR Family Inhibitor. Mol. Cancer Ther..

[91] Subbiah V., Sahai V., Maglic D., Bruderek K., Touré B.B., Zhao S., Valverde R., O’Hearn P.J., Moustakas D.T., Schönherr H. (2023). RLY-4008, the First Highly Selective FGFR2 Inhibitor with Activity across FGFR2 Alterations and Resistance Mutations. Cancer Discov..

[92] Rizzo A., Ricci A.D., Brandi G. (2021). Futibatinib, an investigational agent for the treatment of intrahepatic cholangiocarcinoma: Evidence to date and future perspectives. Expert Opin. Investig. Drugs.

[93] Katoh M. (2019). Fibroblast growth factor receptors as treatment targets in clinical oncology. Nat. Rev. Clin. Oncol..

[94] Facchinetti F., Hollebecque A., Braye F., Vasseur D., Pradat Y., Bahleda R., Pobel C., Bigot L., Déas O., Florez Arango J.D. (2023). Resistance to Selective FGFR Inhibitors in FGFR-Driven Urothelial Cancer. Cancer Discov..

[95] Cowell J.K., Qin H., Hu T., Wu Q., Bhole A., Ren M. (2017). Mutation in the FGFR1 tyrosine kinase domain or inactivation of PTEN is associated with acquired resistance to FGFR inhibitors in FGFR1-driven leukemia/lymphomas. Int. J. Cancer.

[96] Paik P.K., Shen R., Berger M.F., Ferry D., Soria J.C., Mathewson A., Rooney C., Smith N.R., Cullberg M., Kilgour E. (2017). A Phase Ib Open-Label Multicenter Study of AZD4547 in Patients with Advanced Squamous Cell Lung Cancers. Clin. Cancer Res. Off. J. Am. Assoc. Cancer Res..

[97] Coombes R.C., Badman P.D., Lozano-Kuehne J.P., Liu X., Macpherson I.R., Zubairi I., Baird R.D., Rosenfeld N., Garcia-Corbacho J., Cresti N. (2022). Results of the phase IIa RADICAL trial of the FGFR inhibitor AZD4547 in endocrine resistant breast cancer. Nat. Commun..

[98] Kong Y., Zhao X., Wang Z., Yuan S., Chen S., Lou S., Ma S., Li Y., Wang X., Ge Y. (2024). A selective Fibroblast Growth Factor Receptor 1/2 PROTAC degrader with antitumor activity. Mol. Cancer Ther..

